# Febrile or Exanthematous Illness Associated with Zika, Dengue, and Chikungunya Viruses, Panama

**DOI:** 10.3201/eid2208.160292

**Published:** 2016-08

**Authors:** Dimelza Araúz, Luis De Urriola, José Jones, Marlene Castillo, Alexander Martínez, Edison Murillo, Leonidas Troncoso, María Chen, Leyda Abrego, Blas Armién, Juan M. Pascale, Néstor Sosa, Sandra López-Verges, Brechla Moreno

**Affiliations:** Gorgas Memorial Institute for Health Studies, Panama City, Panama (D. Araúz, M. Castillo, M. Chen, L. Abrego, S. López-Verges, B. Moreno, A. Martinez, B. Armién, J.M. Pascale, N. Sosa);; Panama Ministry of Health Department of Epidemiology, Guna Yala, Panama (L. De Urriola);; Health Center Guna Yala, Guna Yala (J. Jones, E. Murillo, L. Troncoso)

**Keywords:** Zika virus, flavivirus, outbreak, dengue virus, chikungunya virus, exanthematous illness, febrile illness, viruses, Panama

**To the Editor:** The earliest clinical cases of Zika virus infection were reported from continental South America in 2015 (*1*), after which the virus spread rapidly through the Americas (*2*). Here we describe an investigation of febrile or exanthematous illnesses for possible association with Zika, dengue, or chikungunya virus; these illnesses occurred in the Guna Yala region of eastern Panama, which borders northern Colombia (Figure).

**Figure:**
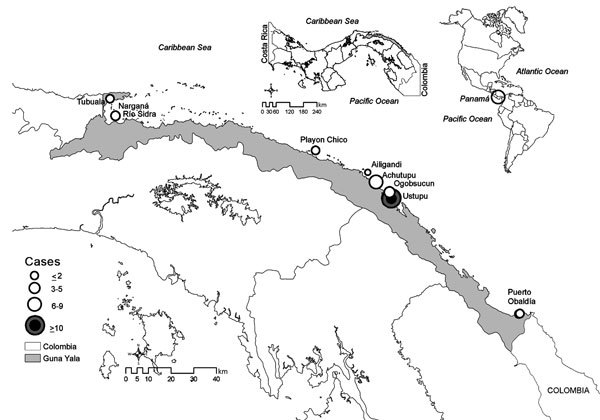
Locations in the Guna Yala region of eastern Panama with confirmed cases of Zika virus infection during November 27, 2015–January 22, 2016. Inset maps show locations of Guna Yala in Panama and of Panama in the Americas.

We collected and analyzed a convenience sample of 276 serum samples and 26 paired urine samples from 276 patients who sought care at clinics in Guna Yala during November 27, 2015–January 22, 2016, for reported fever or rash of <5 days’ duration in addition to 1 of the following: headache, malaise, arthralgia, myalgia, or conjunctivitis. We also collected data on clinical signs and symptoms, date of illness onset, age, sex, residence, and self-reported status of pregnancy.

At first, we performed real-time reverse transcription PCR (rRT-PCR) tests specific for dengue (*3*) and chikungunya (*4*) viruses. However, because all the samples received during the week of November 27 were negative for those viruses and Zika virus was being reported in Colombia as of October 2015, we also tested the samples with a flavivirus-specific rRT-PCR (*5*), followed by amplicon sequencing; or with an rRT-PCR specific for Zika virus (*6*). 

Of the 276 patients whose samples were tested, 164 (60%) were female. A total of 22 (8%) samples were positive for dengue; 2 were positive for chikungunya. Of the remaining 252 patients, 50 (20%) had >1 sample that tested positive for Zika virus (50/252 serum samples, 4/26 paired urine samples). Of these 50 patients, 30 (60%) were female. Most of these patients reported illness onset during December 9–27, 2015 (Technical Appendix Figure 1). Zika virus infection affected all age groups (median age 35 y, range 0.1–80 y). 

The most commonly reported signs and symptoms were fever (86%), exanthema (72%), and headache (62%). The clinical characteristics of these infections showed no statistically significant difference with those associated with dengue and chikungunya virus infections and with cases found to be negative for all 3 viruses, suggesting that the negative cases could represent Zika virus infections (Technical Appendix Table). One of the patients with confirmed Zika virus infection reported being in her second trimester of pregnancy; she underwent a fetal ultrasound at 36 weeks’ gestation, which was interpreted as normal, and the infant was found to have no neurologic defects at birth.

By using Vero E6 cells (American Type Culture Collection), we isolated Zika virus from 9 samples (8 serum, 1 urine). Phylogenetic analysis of 5 Zika virus sequences (a 428-nucleotide fragment encompassing a conserved region of the nonstructural protein 5 gene) placed these isolated (GenBank accession nos. KU724096–100) within the Asian lineage, the lineage involved in the spread of Zika virus in the Americas (Technical Appendix Figure 2) (*2*,*7*).

By using molecular methods, we confirmed diagnoses in 27% of patients during this outbreak. The distribution of positive results suggests that Zika virus was the predominant etiologic virus in this cohort, but we cannot firmly conclude this because most specimens tested negative for Zika, dengue, and chikungunya viruses.

Although results from patient sampling and laboratory testing are not comparable, an assessment in Puerto Rico was able to detect Zika virus RNA by rRT-PCR or IgM by ELISA in 19% of 155 patients with suspected Zika virus infection (*8*). Despite the addition of IgM testing, most of the patients whose specimens were tested by rRT-PCR were negative for dengue and Zika viruses.

Several reasons might exist for the high proportion of specimens testing negative for Zika virus. Viremia is often low and short-lived in persons infected with Zika virus (*7*); the PCR test might not be sensitive enough; some patients with Zika virus infection may have sought care after the virus had been cleared from the blood and urine; our diagnostic capacity was limited by the lack of reliable serologic tests for Zika virus; and we did not test for other viral, bacterial, or parasitic causes of fever or rash illness.

The Panama Ministry of Health is following up with known pregnant women of the Guna Yala region who report Zika virus infection symptoms and is testing urine samples by using Zika virus–specific rRT-PCR within 14 days of symptom onset. Pregnant women confirmed to have Zika virus infection will receive ultrasound monitoring; however, the test has relatively low positive predictive value for detecting microcephaly (*9*). In Guna Yala, no symptoms of Guillain-Barré syndrome or other neurologic conditions have been detected; however, since January 2016, Zika virus has spread to other regions of Panama, and at least 1 case of Guillain-Barré syndrome has been reported (*10*). Our experience shows the challenge of diagnosing the causes of fever or rash by using only molecular methods, underscoring the need for diagnostic tools that are rapid and inexpensive but more sensitive and specific.

Technical Appendix Distribution of symptoms by patients with Zika, dengue, and chikungunya virus infections in the Guna Yala region of Panama, November 27, 2015–January 22, 2016; epidemiologic curve for the outbreak of Zika virus infection in this region; and maximum-likelihood tree of the Zika virus isolated during the outbreak.
