# Supplementary material for: Febrile or Exanthematous Illness Associated with Zika, Dengue, and Chikungunya Viruses, Panama
Source: Emerg Infect Dis. 2016 Aug;22(8):1515–7. doi: 10.3201/eid2208.160292 (PMC4982178; doi:10.3201/eid2208.160292)
Supplement: Technical Appendix — Distribution of symptoms by patients with Zika, dengue, and chikungunya virus infections in the Guna Yala region of Panama, November 27, 2015–January 22, 2016; epidemiologic curve for the outbreak of Zika virus infection in this region; and maximum-likelihood tree of the Zika virus isolated during the outbreak. [file 16-0292-Techapp-s1.pdf]

Article DOI: http://dx.doi.org/10.3201/eid2208.160292

Febrile or Exanthematous Illness Associated with Zika, Dengue, and Chikungunya Viruses, Panama

Technical Appendix

Technical Appendix Table. Distribution of symptoms among patients with confirmed cases of Zika (n = 50), dengue (n = 22), chikungunya (n = 2) virus infections and those found to be negative for all 3 viruses (n = 202), Guna Yala region, Panama, November 27, 2015–January 22, 2016*

Symptom	Zika, n (%)	Dengue, n (%)	Chikungunya, n (%)	Negative, n (%)
Fever	43 (86)	20 (91)	1 (50)	181 (90)
Exanthema	36 (72)	14 (64)	2 (100)	159 (79)
Headache	31 (62)	11 (50)	1 (50)	110 (54)
Arthralgia	20 (40)	6 (27)	1 (50)	58 (29)
Myalgia	17 (34)	8 (36)	1 (50)	59 (29)
Chill	12 (24)	7 (32)	0	51 (25)
Retroorbital pain	7 (14)	5 (23)	0	28 (14)
Conjunctivitis	5 (10)	1 (5)	0	32 (16)
Diarrhea	3 (6)	1 (5)	0	3 (1)
Petechial rash	1 (2)	0	0	3 (1)

*Statistical analysis were done using chi-square or Fisher's exact test. No statistical differences were found between Zika and dengue, Zika and negative, or dengue and negative.

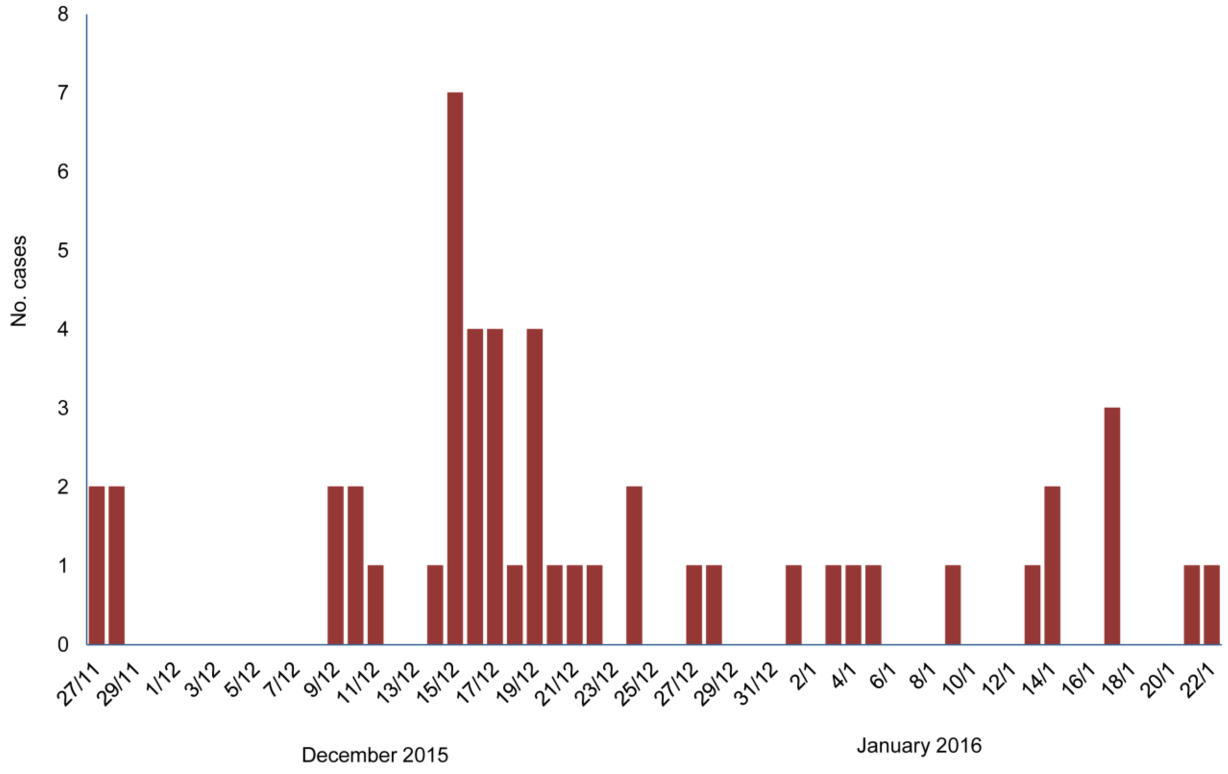

Technical Appendix Figure 1. Epidemiologic curve of the number of confirmed cases of Zika virus infection per day, by date of illness onset.

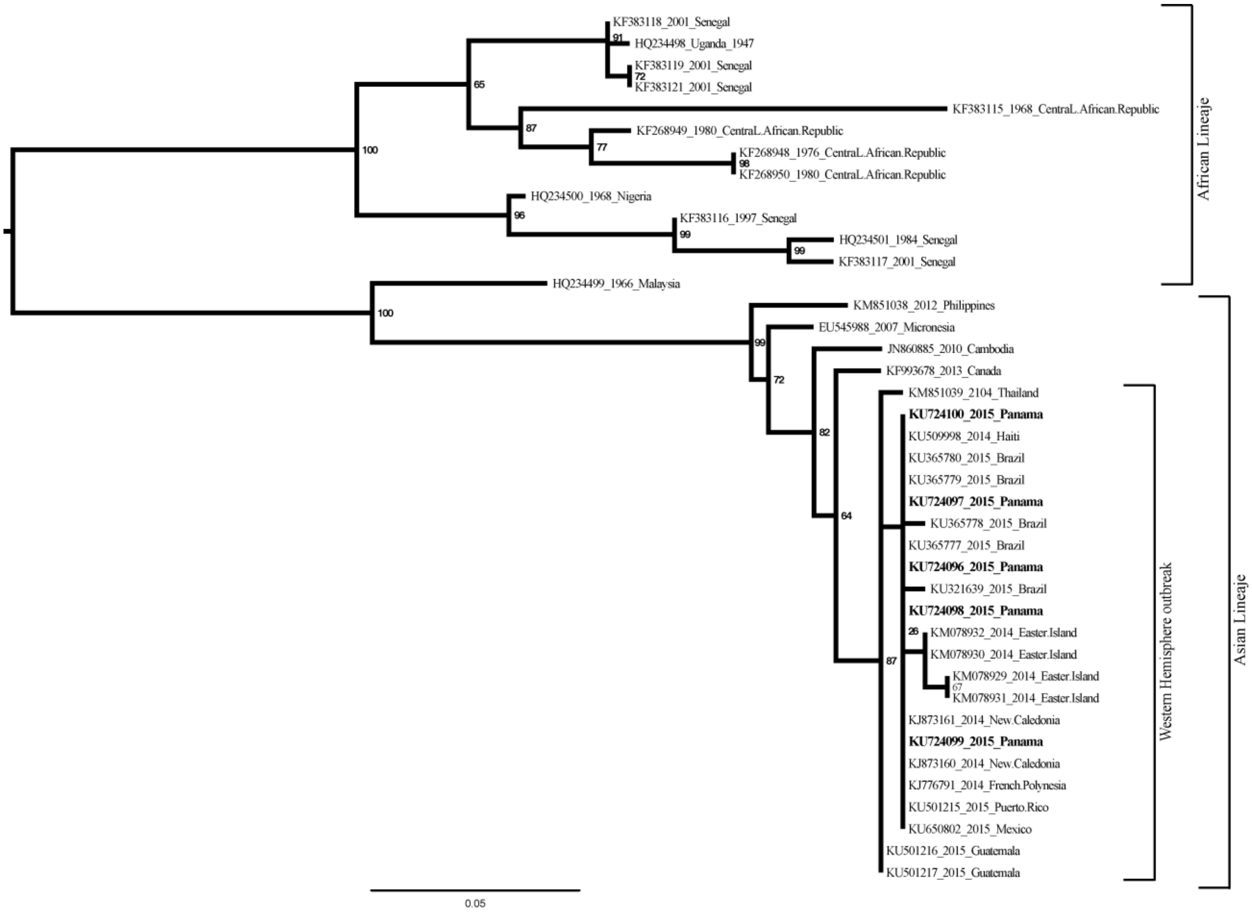

Technical Appendix Figure 2. Maximum-likelihood tree of the Zika virus isolated from the Guna Yala outbreak in Panama, based in a partial fragment (~428bp) of the nonstructural protein 5 gene of Zika virus. Panama samples are highlighted. Tree is midpoint rooted. The tree was inferred under TN93 +G6 substitution model selected by smart model selection as is implemented in PhyML 3.0 beta version. The tree was reconstructed with PhyML. The tree reliability topology was estimated using bootstrap resampling (1,000 replicates). Tree was visualized with FigTree v1.4.2. A clustering (bootstrap value >85%) is observed among the sequences published from the ongoing outbreak in the Americas, suggesting that a common genotype, within the Asian lineage, is involved.
